# Whole-Transcriptome Analysis of LncRNAs Mediated ceRNA Regulation in Granulosa Cells Isolated From Healthy and Atresia Follicles of Chinese Buffalo

**DOI:** 10.3389/fvets.2021.680182

**Published:** 2021-07-14

**Authors:** Yu Pan, Sufang Yang, Juanru Cheng, Qiao Lv, Qinghua Xing, Ruimen Zhang, Jingyuan Liang, Deshun Shi, Yanfei Deng

**Affiliations:** State Key Laboratory for Conservation and Utilization of Subtropical Agro-Bioresources, Animal Reproduction Institute, Guangxi University, Nanning, China

**Keywords:** buffalo (*Bubalus bubalis*), follicles, granulosa cells, whole-transcriptome, ceRNA

## Abstract

Granulosa cells (GCs) are the main supporting cells in follicles and play an important role in the regulation of oocyte maturation and follicular atresia. Accumulating evidence indicates that non-coding RNAs participate in regulation of the physiological function of GCs. However, whole-transcriptome analysis for GCs of buffalo has yet to be reported. In this study, healthy follicles (HFs) and atretic follicles (AFs) were defined according to the apoptosis rate of GCs and the hormone level in follicular fluid. GCs were collected from HFs and AFs (*n* = 15, 5 < *n* < 8 mm) for whole-transcriptome analysis using second-generation high-throughput sequencing. A total of 1,861 and 1,075 mRNAs, 159 and 24 miRNAs, and 123 and 100 lncRNAs, were upregulated and downregulated between HFs and AFs, respectively. Enrichment of functions and signaling pathways of these differentially expressed (DE) genes showed that most of DEmRNAs and targets of DEmiRNAs were annotated to the categories of ECM–receptor interaction and focal adhesion, as well as PI3K-AKT, mTOR, TGF-beta, Rap1, and estrogen signaling pathways. The competing endogenous RNA (CeRNA) network was also constructed based on the ceRNA theory which further revealed regulatory roles of these DERNAs in GCs of buffalo follicles. Finally, we validated that lnc4040 regulated the expression of *Hif1a* as miR-709 sponge in a ceRNA mechanism, suggesting their critical functions in GCs of buffalo follicles. These results show that lncRNAs are dynamically expressed in GCs of HFs and AFs, and interacting with target genes in a ceRNA manner, suggesting their critical functions in buffalo follicular development and atresia.

## Introduction

In the process of mammalian follicle development, more than 99% of follicles become atretic and very few of follicles develop to ovulation (1). Chinese buffalo is an important large domestic animal distributed in the tropical and subtropical regions of China. It is generally believed that the sexual maturity of buffalo is later, and its postpartum estrus period is longer than that of cattle (2, 3). As a mono-ovulating animal, follicular atresia is also an important factor restricting its reproductive performance, and the follicular atresia rate of buffalo is higher than that of cattle (4). During each estrus period, only one follicle matures and ovulates and other follicles undergo atresia during follicular development, and this occurs repeatedly throughout the developmental stages. However, the exact mechanism of cell death during this process is not fully understood. Therefore, elucidating the molecular mechanism of follicle atresia is expected to reverse the fate of follicles in buffalo, thus effectively utilizing more follicle resources and improving the reproductive efficiency of Chinese buffalo.

Previous studies have shown that follicular atresia is closely related to the apoptosis of ovarian granulosa cells (GCs) (5–8). GCs gradually shrink in size, and apoptotic bodies are formed. The chromatin is then condensed, a large amount of which is shed into the follicular fluid of atretic follicles (AFs) and the blood supply around the follicles is greatly reduced (9–12). In addition, interferon and some growth factors can also induce apoptosis. The intrinsic factors related to apoptosis are usually those associated with stress, such as nutritional deficiency, oxidative damage, and genetic damage as well as molecular stress (13). Recent studies have shown that the autophagy of GCs partially dominates the occurrence of follicular atresia (14–16).

However, the molecular mechanism of follicular atresia needs to be further explored. The development of transcriptome techniques provides an effective route to study the mechanism of follicular development and atresia. Early transcriptomics studies of follicles focused on mRNA, and researchers conducted studies at different stages of follicular development in cattle (17–20). Subsequent research showed that miRNA expression was spatiotemporal specific during bovine follicular development. In contrast to small follicles, bta-mir-144, bta-mir-202, bta-mir-451, bta-mir-652, and bta-mir-873 were all upregulated in large healthy follicles (HFs) and they targeted the signaling pathways involved in follicular cell proliferation and steroid generation (21). Long non-coding RNAs (lncRNAs) have received considerable attention for their important roles in epigenetic regulation, chromatin modification, genomic imprinting, transcriptional control, and pre-translational as well as posttranslational mRNA processing (22, 23).

The main mechanism of competing endogenous RNAs (ceRNAs) is to competitively bind miRNAs, remove the inhibitory effect of miRNAs on target genes, and improve the expression levels of target genes (24). The development of this hypothesis has given mRNAs and non-coding RNAs new and broader biological functions (25). The research between compact cumulus cells and expanded GCs found 89 lncRNAs, of which 12 are encoded within introns of genes known to be involved in GC processes. This suggested that unique non-coding RNA transcripts may contribute to the regulation of cumulus expansion and oocyte maturation (26).

In the study of mammalian follicles, researchers have obtained a large amount of transcriptomics data, which has a crucial regulatory role at all stages of follicular development (27). Several miRNAs and lncRNAs were screened for specific signal pathway regulation analysis. However, for large domestic animals, there are few such studies. Therefore, we used high-throughput sequencing to perform a whole-transcriptome analysis of GCs isolated from AFs and HFs of the Chinese buffalo, in order to obtain various types of DE-RNA molecules, and this allowed the construction of a ceRNA network to explore the molecular regulatory pathways involved. The non-coding RNAs obtained in this study can provide a strong basis for the subsequent study of the altered physiological functions of GCs during follicular development and atresia in buffalo. The aim of the study was to provide a theoretical basis and clues for follicular atresia observed in the Chinese buffalo.

## Results

### Appearance and Classification of HFs and AFs in Buffalo

**Figure 1:**
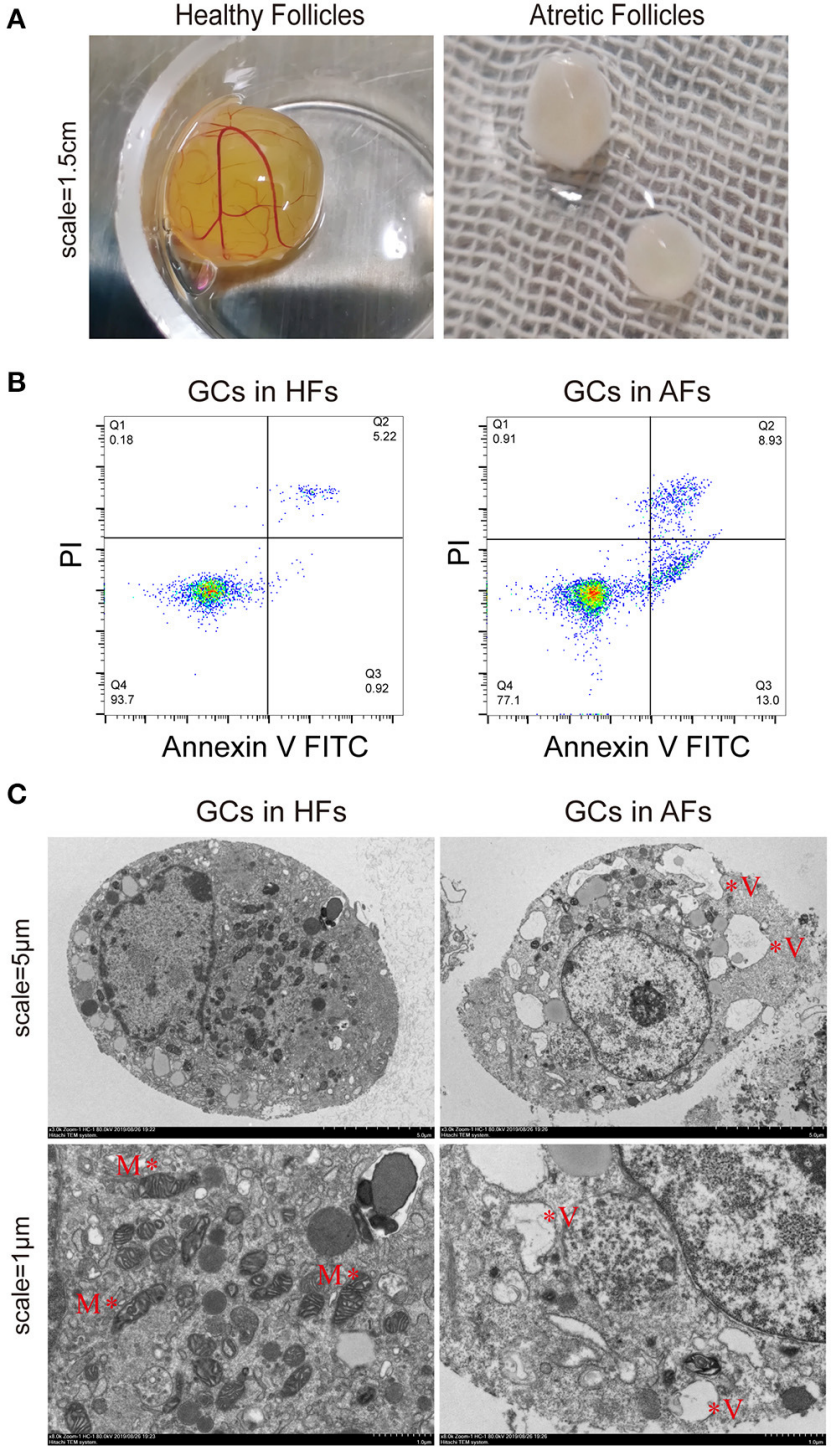
Phenotypic detection of healthy and atretic follicles. **(A)** The appearance of follicles with scale bars = 1.5 cm: the capillaries were rich on the surface of healthy follicles, and the follicular fluid was yellow, while little or no capillaries were seen on the surface of atretic follicles, and the follicular fluid was grayish white. **(B)** The apoptosis rate of the granulosa cells (*n* = 3) of healthy and atretic follicles was assessed by flow cytometry. **(C)** Transmission electron microscope of granulosa cells in healthy and atretic follicles with scale bars = 5 and 1 μm, respectively. M indicates mitochondria, and V represents vacuoles.

By observing exposed follicles, the surfaces of HFs were seen to be rich in capillaries and the follicular fluid was luminous yellow, while little or no capillaries were observed on the surface of AFs, and the follicular fluid was grayish white (Figure 1A). Follicular fluid and GCs were isolated from both HFs and AFs. The concentrations of estradiol (E_2_) and progesterone (PROG) in isolated follicular fluid were measured by ELISA, and these were 126.21 ± 9.28 and 21.88 ± 1.78 ng·ml^−1^, respectively, and the ratio was 5.77. The concentrations of E_2_ and PROG in AFs were 76.63 ± 1.09 and 53.43 ± 2.68 ng·ml^−1^, respectively, and the ratio was 1.43 (Table 1). The apoptosis rate of GCs in HFs was 4.93 ± 1.59, while that in AFs was 21.31 ± 1.40 (Figure 1B and Table 2). Transmission electron microscope observations showed that the morphology and submicrostructure of each organelle in HF were more normal than those in AF, there were more mitochondria in HF compared to the AF, chromatin breaks down to form granules, and a large number of vacuoles and apoptotic bodies appear in the cytoplasm of GCs in AFs (Figure 1C).

**Table 1:** Hormone levels in follicular fluid of HFs and AFs.

**Follicle classification**	**Estrogen content (ng·ml^**−1**^)**	**Progesterone content (ng·ml^**−1**^)**	**E_**2**_/PROG (per)**	**E_**2**_/PROG**
Healthy follicles (*n* = 6)	133.83	25.74	5.20	5.90 ±^a^
	124.43	23.99	5.19	
	107.76	18.29	5.89	
	167.78	27.42	6.12	
	115.70	17.55	6.59	
	125.74	19.66	6.40	
Atretic follicles (*n* = 5)	75.24	55.79	1.35	1.35 ±^b^
	72.83	48.93	1.49	
	76.59	49.07	1.56	
	79.26	62.93	1.26	
	82.20	74.36	1.11	

*The data are the means ± SEM*.

a, b *Indicate p < 0.01 for HFs and AFs, respectively*.

**Table 2:** Apoptotic rate of GCs in HFs and AFs.

	**GCs of HFs**			**GCs of AFs**		
Q1 (%)	0.18	0.07	0.13	0.91	3.60	2.30
Q2 (%)	5.22	5.77	0.63	8.93	5.84	21.50
Q3 (%)	0.92	1.11	1.14	13.00	12.80	1.86
Q4 (%)	93.70	93.00	98.10	77.10	77.70	74.30
Q2+Q3	6.14	6.88	1.77	21.93	18.64	23.36
Apoptosis rate (%)	4.93 ± 1.59^a^			21.31 ± 1.40^b^		

*The data are the means ± SEM, n = 3*.

a, b *Indicate p < 0.01 for HFs and AFs, respectively*.

### Overview of mRNA-Seq Data

**Figure 2:**
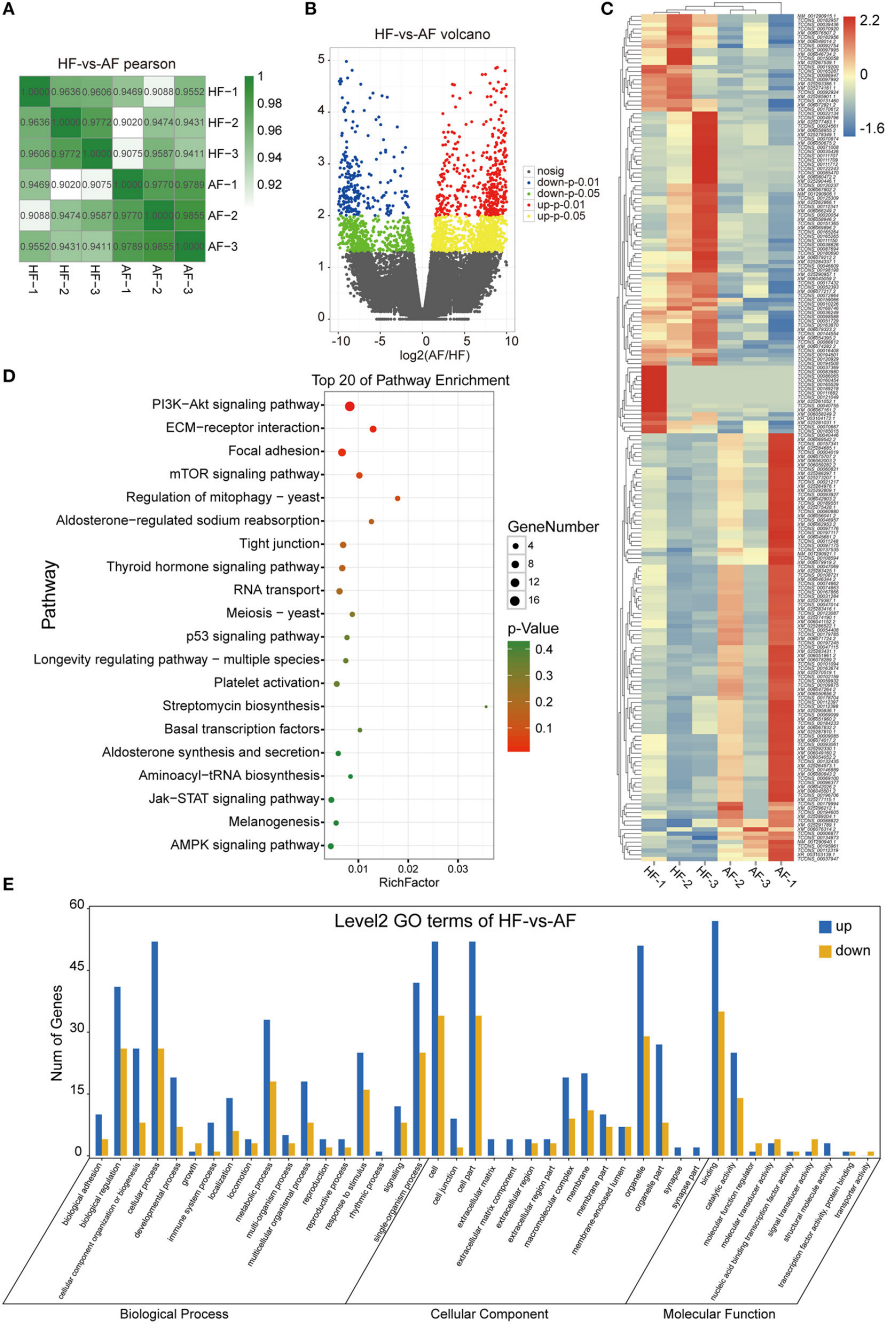
Identification and analysis of DEmRNAs in granulosa cells between healthy and atretic follicles. **(A)** The Pearson correlation heat map between each sample. **(B)** Volcano plot diagrams showing log2FC and p-values of DEmRNAs. **(C)** Clustering heat map for DEmRNAs in each sample between the two groups. The expression of DEmRNAs was calculated with 2 as the base logarithm, and different samples and transcripts were analyzed by hierarchical cluster analysis; red and blue represent upregulation and downregulation, respectively. **(D)** Top 20 of the KEGG pathway bubble chart of DEmRNAs. The bubble size represents the number of mRNAs enriched in the pathway, and the bubble color represents the *p*-value. **(E)** GO enrichment histogram of DEmRNAs; blue and yellow represent upregulation and downregulation, respectively.

From mRNA-seq data, 40,990 protein-coding genes were identified in the buffalo genome. The Pearson correlation coefficient calculated according to the gene fragments per kilobase of exon model per million reads mapped (FPKM) between the HFs and AFs samples showed that the consistency among the three repeated samples in the same group was more than 0.9, indicating that the repeatability between each two samples was dependable (Figure 2A). Their log2FC values are presented as volcano plot diagrams in Figure 2B. Among all of these genes, 2,936 (1,861 upregulated and 1,075 downregulated) DEmRNAs (fold change ≥ 2 and *p*-value < 0.05) were identified in GCs between HFs and AFs. A heat map of DEmRNAs showed the general expression profiles of the DEmRNAs in each group (Figure 2C).

To explore the functions of the DEmRNAs, KEGG was the main public database used regarding the pathway. The results showed that there were 42 and 72 DEmRNAs significantly enriched to ECM–receptor interactions and focal adhesion, respectively. In addition, there were 102, 48, 29, 35, and 33 enriched to the PI3K-AKT, thyroid hormone, TGF-beta, mTOR, and estrogen signaling pathways, respectively (Figure 2D). GO annotation and GO enrichment analysis were also performed. Based on GO annotation, it was found that DEmRNAs were annotated to the cellular process, biological regulation, single-organism processes, metabolic processes, and response to stimulus under biological processes (BP), to the cells, cell parts, organelles, and organelle parts under cellular components (CC) and to the binding, catalytic activities, and molecular transducer activities under molecular functions (MF) (Figure 2E). In organisms, different transcripts coordinated with each other to exercise their biology and pathway-based analysis is helpful to further understand the biological functions of the transcripts.

### Overview of miRNA-Seq Data

**Figure 3:**
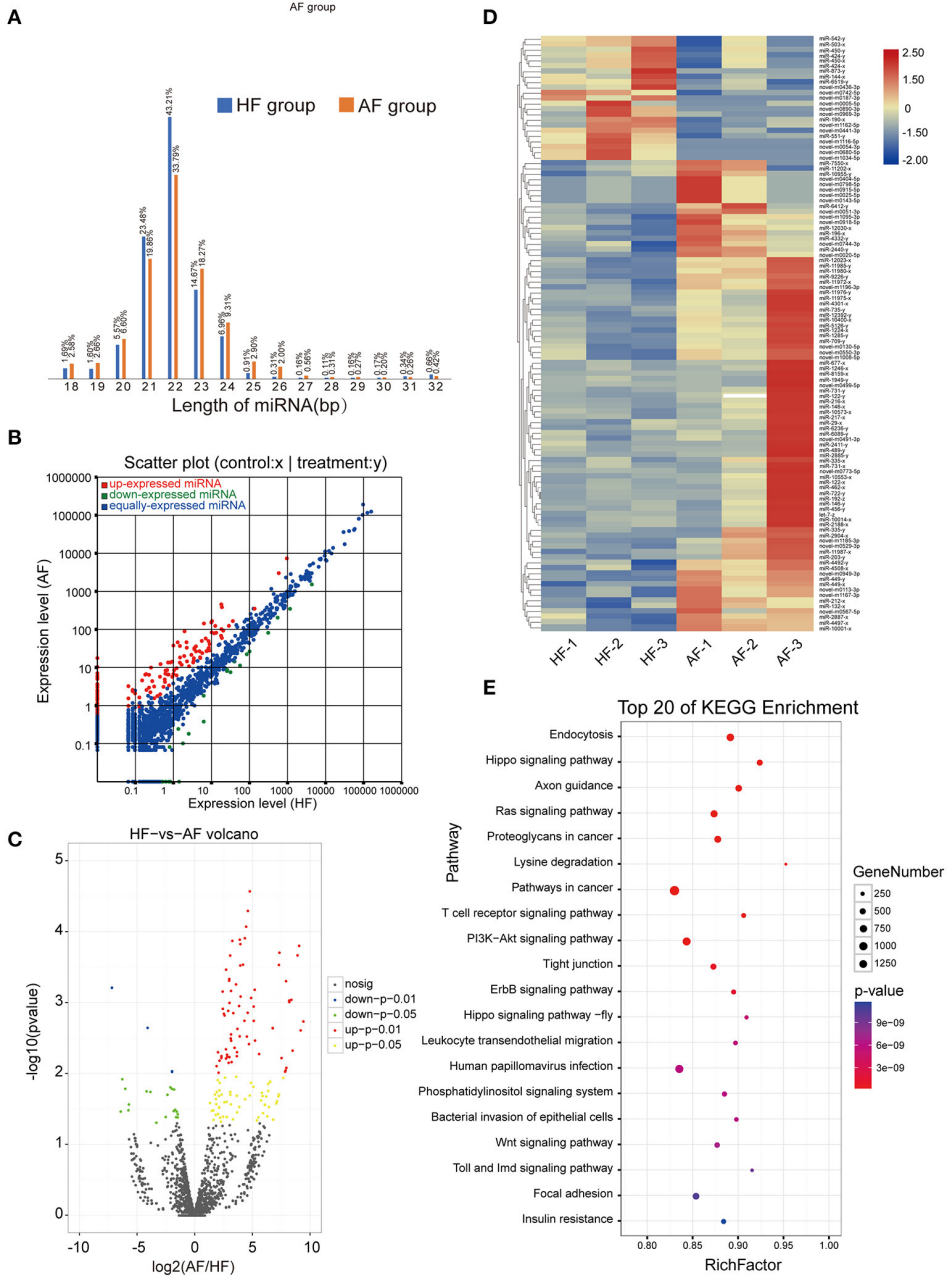
Identification and analysis of DEmiRNAs in granulosa cells between healthy and atretic follicles. **(A)** Length statistic histogram of tags between the two groups. **(B)** The expression of identified miRNAs between the two groups was upregulated (30) and downregulated (blue). **(C)** Volcanic diagram of the expression of DEmiRNAs in different groups. **(D)** Clustering heat map for DEmiRNAs in each sample between the two groups with red for upregulation and blue for downregulation. **(E)** Top 20 of the KEGG pathway bubble chart of target mRNAs; the bubble size represents the number of mRNAs enriched in the pathway, and the bubble color represents the *p*-value.

A total of 704,220 tags were generated. The results were filtered based on length (18–35 nt), and most selected tags were 22 nt in length in both the HF and AF groups (Figure 3A). All the clean tags were aligned with the reference genome. The tags mapped to repeat sequences were removed. Ultimately, 2,446 mature miRNAs (1,173 known and 1,273 novel) were detected, and these were drawn as a scatter plot using the expression between the different groups (Figure 3B). We identified miRNAs with a fold change ≥ 2 and *p*-values < 0.05 in a comparison of significant DEmiRNAs (159 upregulated and 24 downregulated) (Figure 3C). The heat maps of all DEmiRNAs were drawn to display the miRNA expression levels in different samples and to cluster miRNAs with a similar expression pattern (Figure 3D). Targeted mRNAs of these DEmiRNAs are listed in Supplementary Table 1. Pathway enrichment analysis of the targets of DEmiRNAs showed that there were 930, 884, 702, and 407 mRNAs linked to PI3K-Akt, MAPK, Ras, and mTOR signaling pathways, respectively. In addition, 799 and 400 mRNAs were linked to endocytosis and autophagy, respectively (Figure 3E).

### Overview of lncRNA-Seq Data

**Figure 4:**
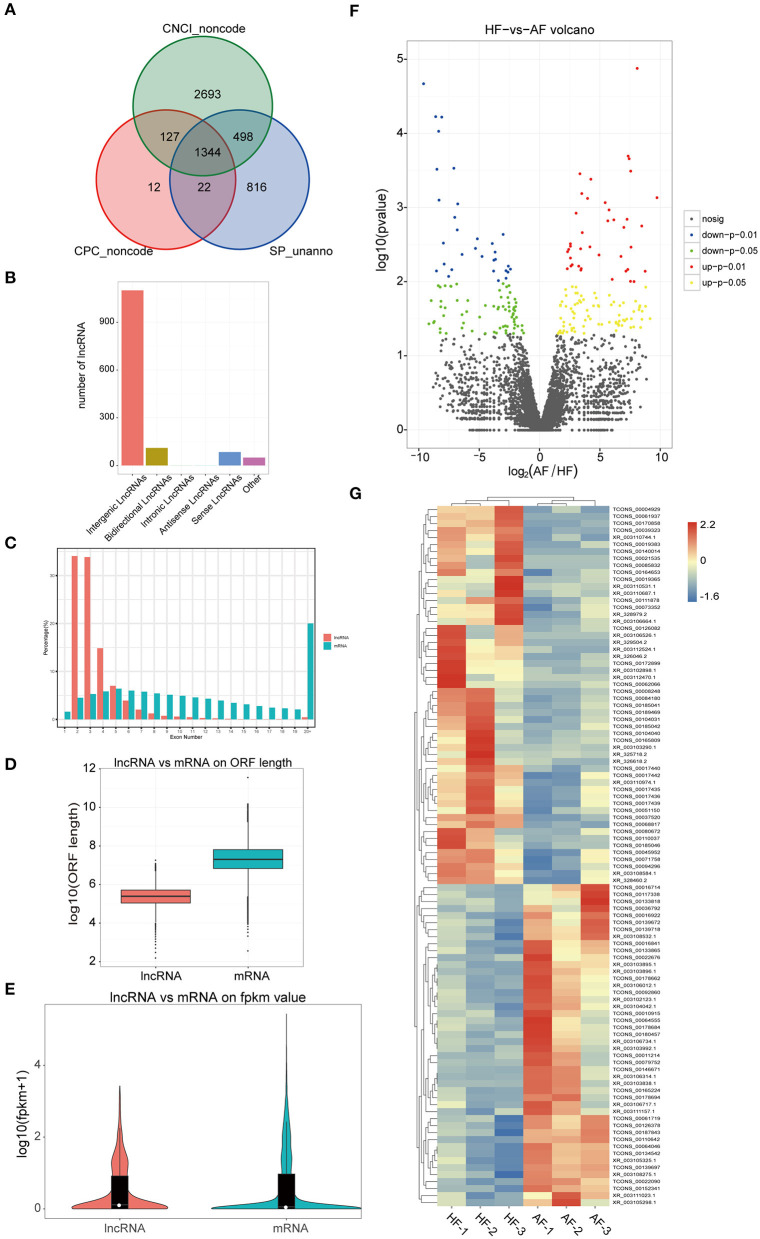
Identification and analysis of DElncRNAs in granulosa cells between healthy and atretic follicles. **(A)** Venn diagram of the lncRNA annotation results of CPC, CNCI, and SwissProt. **(B)** LncRNA classification histogram. **(C–E)** Comparison of lncRNA with mRNA with respect to the transcript length, exon number, ORF length, and FPKM values. **(F)** Volcano plot of the expression of DElncRNAs in different groups. **(G)** Heat map of all DElncRNAs.

The expressions of 3,488 known and 1,338 novel transcripts were identified. Two different softwares, namely, CNCI (version 2) (28) and CPC (29) (http://cpc.cbi.pku.edu.cn/), were used to assess the protein-coding potential of novel transcripts by default parameters. Novel transcripts were mapped with the SwissProt database to assess protein annotation. The intersection results of both non-protein-coding potential and non-protein annotation were chosen as the new lncRNAs (1,344) (Figure 4A). The lncRNAs obtained were classified into five classes according to their location relative to protein-coding genes, and these were named intergenic, bidirectional, intronic, antisense, and sense overlapping lncRNAs. The different types of lncRNAs probably have different biological functions (Figure 4B). A comparison of the genomic characterizations of the lncRNAs with mRNAs showed that their transcripts were similar in length distribution, except that lncRNA had relatively higher numbers of long transcripts (>4,500 bp) than mRNAs. For the number of exons, a higher percentage of lncRNAs had two to four exons. In addition, lncRNAs tended to have a shorter ORF length and a lower FPKM value (Figures 4C–E). Transcripts with a fold change ≥ 2 and a *p*-value < 0.05 were identified in a comparison as significant DElncRNAs (209 in final, 122 upregulated, and 87 downregulated) (Supplementary Table 4) were seen when a volcano plot was drawn (Figure 4F). Based on the expression of the DElncRNAs, the relationship between the sample and the DElncRNAs was clustered hierarchically, and a heat map is used to represent the clustering results (Figure 4G).

### CeRNA Regulatory Network in GCS Between HFs vs. AFs

To reveal the global regulatory network of protein-coding RNAs and ncRNAs in GCs between HFs vs. AFs, a ceRNA network was constructed using DEmiRNAs, DEmRNAs, and DElncRNAs based on the ceRNA theory. In total, 1,458 DEmRNAs and 134 DElncRNAs were predicted as targets for 135 miRNAs. The lncRNA–miRNA–mRNA network was constructed by assembling all the co-expression competing triplets, which has been identified above. The connectivity of RNA in the ceRNA network was defined as the number of co-expressed targeted miRNAs. So ceRNAs with the highest connectivity were regarded as hub genes, which are more essential in biological networks.

In this ceRNA network, miR-424-3p, miR-542-5p, miR-735-5p, mi-503-3p, miR-212-3p, miR-216-3p, and miR-2440-5p are involved in morethan 100 nodes, suggesting that they may act as core regulators. In addition, lncRNAs including XR_003111160.1, XR_003106664.1, XR_003103086.1, XR_327919.2, XR_003111331.1, XR_003103012.1, XR_003111622.1, and TCONS_00165726 participated in the network.

**Figure 5:**
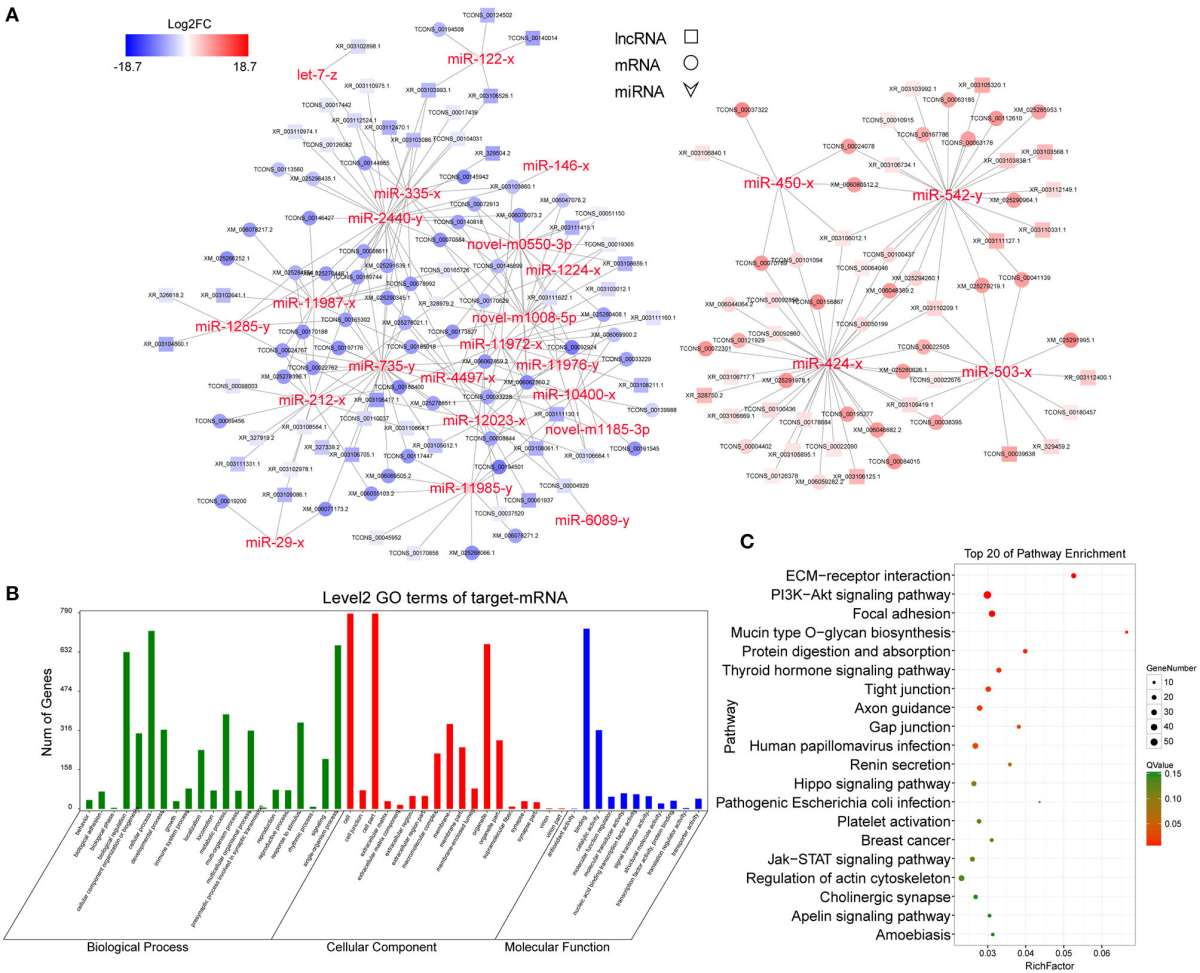
CeRNA regulatory network and functional analysis. **(A)** CeRNA network built with the top 30 DEmiRNAs. Red and blue represent the upregulated and downregulated levels, respectively. **(B)** Level 2 GO enrichment of target mRNAs. **(C)** Top 20 KEGG pathways of target mRNAs.

Because of the large number of node genes and the tedious gene interaction network, the top 30 DEmiRNAs were selected as the source genes to simplify visualization using Cytoscape software (v3.6.0) (http://www.cytoscape.org/) (Figure 5A) with their target mRNAs. These 30 miRNAs included miR-146-3p, miR-450-3p, miR-424-3p, miR-212-3p, and miR-29-3p. GO enrichment analysis of all target mRNAs showed that most mRNAs were enriched in cell parts, binding, cellular processes, and organelles. KEGG pathways were enriched in ECM–receptor interactions, PI3K-AKT signaling pathway, protein digestion and absorption, focal adhesion, Hippo signaling pathway, mTOR signaling pathway, and various other metabolic pathways (Figures 5B,C).

### Verification of the Regulation of *Hif1a* by ceRNA Circuitry in GCs

**Figure 6:**
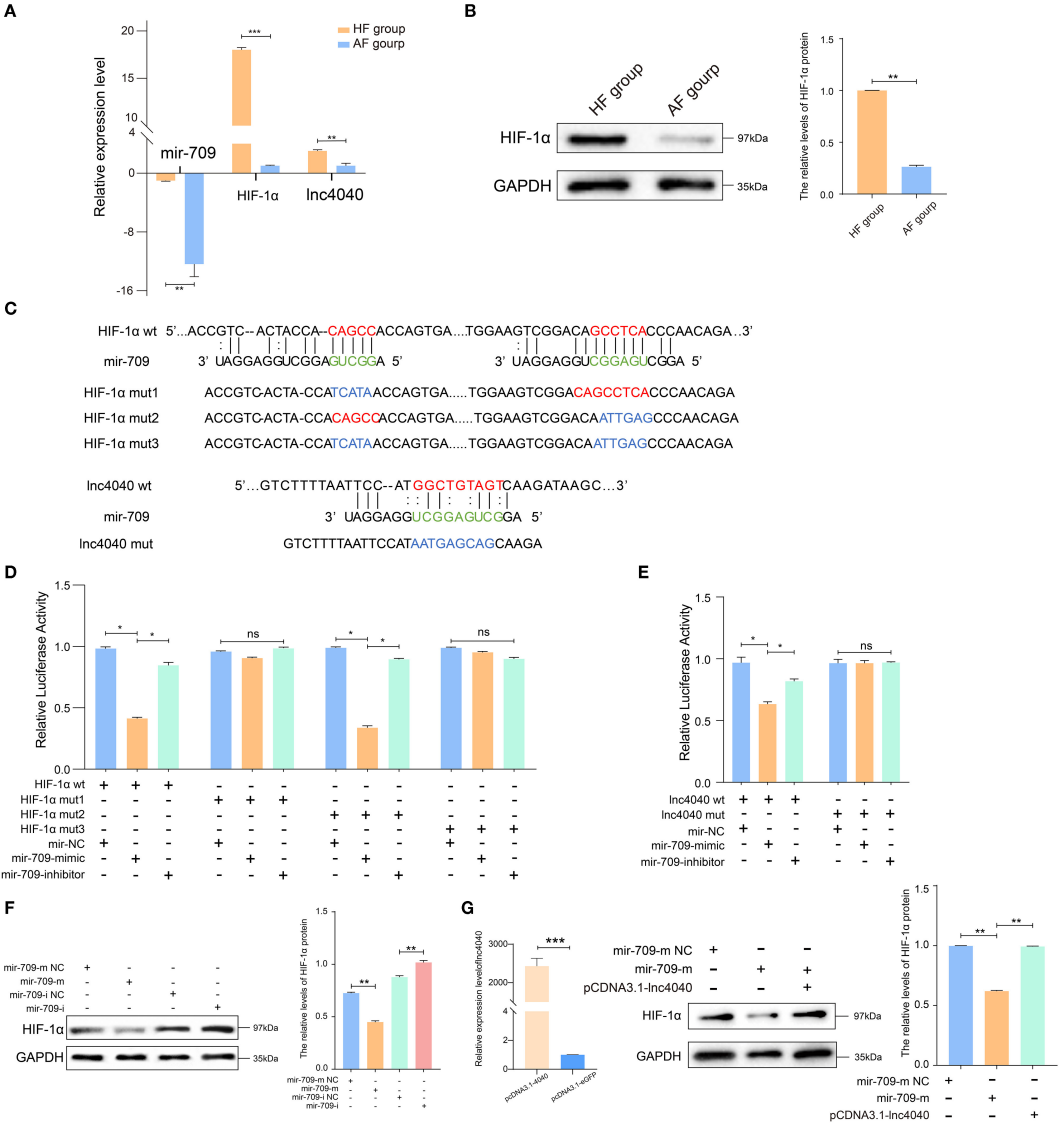
Experimental validated of ceRNAs regulation with miR-709. **(A)** Relative expression levels of miR-709, *Hif1a*, and lnc4040 in GCs between HFs and AFs. **(B)** Expression of HIF1A protein in GCs between HFs vs. AFs and quantified by ImageJ (v1.45), error bars represent SEM (*n* = 3) **p < 0.01. **(C)** Predicted miR-709-binding site on *Hif1a* and lnc4040, and design of luciferase reporter. **(D)** 293T cells were co-transfected with wild-type (WT) or mutant (MUT) luciferase reporters of *Hif1a* with miR-709 mimics, mimics NC, or miRNA inhibitors. The relative levels of firefly luminescence normalized to Renilla luminescence are plotted. Error bars represent SEM (*n* = 3) *p < 0.05. **(E)** 293T cells were co-transfected with wild-type (WT) or mutant (MUT) luciferase reporters of lnc4040 with miR-709 mimics, mimics NC, or miRNA inhibitors. The relative levels of firefly luminescence normalized to Renilla luminescence are plotted. Error bars represent SEM (*n* = 3) *p < 0.05. **(F)** Expression of HIF1A protein in GCs infected with miR-709 mimics, mimics NC, miR-709 inhibitor, and inhibitor NC and quantified by ImageJ (v1.45); error bars represent SEM (*n* = 3) **p < 0.01. **(G)** Expression of HIF1A protein in GCs infected with miRNA mimics, mimics NC, and lnc4040 overexpression vector and quantified by ImageJ (v1.45); error bars represent SEM (*n* = 3) **p < 0.01.

To further support the ceRNA hypothesis, a regulatory circuitry containing TCONS_00104040 (lnc4040), miR-709-5p (miR-709), and TCONS_00111150 (*Hif1a*) was selected for further verification. *Hif1a* can regulate the proliferation, autophagy, and cell cycle of GCs, and it also plays an important role in ovulation selection (31–33). Our initial experiments confirmed that the expression of lnc4040 was positively correlated with *Hif1a* and negatively correlated with miR-709; Western blot analysis revealed that the protein expression of *Hif1a* was significantly higher in the HF group than in the AF group (Figures 6A,B). We hypothesized that lnc4040 function as miRNA (miR-709) sponges to regulate the expression of *Hif1a* indirectly. To further confirm the binding events among miR-709, lnc4040, and *Hif1a*, we predicted the potential miR-709-binding site within lnc4040 and *Hif1a* by RNA22 version 2.0 (http://cm.jefferson.edu/rna22/Interactive/) (Figure 6C). Subsequent experiments validated the direct regulatory effect of miR-709 on *Hif1a* in 293T cells. Dual luciferase expression vectors carrying the wild-type (WT) miR-709-binding sites or one of the mutated versions of the miR-709-binding sites were constructed. MiR-709 mimics by co-transfection inhibited luciferase activity by the luciferase vectors carrying the mutations in site 1 (mut-1) or both sites 1 and 2 (mut-3), but not by the luciferase vectors carrying the WT *Hif1a*-binding sites or the mutation in binding site 2 (mut-2) (Figure 6D). This suggests that the binding site 1 of *Hif1a* might play the major role in miR-709 regulation of *Hif1a*. We also found that miR-709 can significantly inhibit the luciferase activity (Figure 6E). The HIF1A protein expression level in cultured GCs was decreased after miR-709-mimic transfection and conversely was increased after miR-709-inhibitor transfection (Figure 6F). Moreover, after overexpression of lnc4040, the downregulation of HIF1A protein expression induced by miR-709-mimic was alleviated (Figure 6G). These results evidenced that lnc4040 may function as a sponge of miR-709 to regulate the expression of *Hif1a*. In summary, our results showed that three-node lncRNA-mediated regulatory circuitry might play an essential role in GCs during follicle development.

## Materials and Methods

### Tissues

All experiments regarding animals were performed in the State Key Laboratory for Conservation and Utilization of Subtropical Agro-bio-resources and were conducted in accordance with its guidelines for the care and use of laboratory animals. Ovaries were collected from a local abattoir in Nanning (from non-pregnant female buffaloes) and transported to the laboratory in sterile saline maintained at 38°C. After rinsing with 75% ethanol, the ovaries were placed on a gauze after high-pressure treatment, then sliced open, and single follicles (5 < *n* < 8 mm, *n* = 20) were separated from the ovarian tissue with ophthalmic scissors and repeatedly rinsed with 0.1 M phosphate buffer (pH 7.25).

In this experiment, a total of 15 follicles were successfully collected, and eight HFs and seven AFs were identified. Because a certain amount of follicular fluid is needed to detect hormone levels, we selected six HFs and five AFs for testing. Since the GCs collected need to be detected for apoptosis rate, electron microscopy, and subsequent RNA sequencing, we only collected enough GCs from three HFs and three AFs for experiments.

### Classification of HFs and AFs

We judged the status of a follicle by the appearance of the follicle. We classified the follicles with yellowish surfaces, rich capillaries and they bright red color, uniform distribution, meanwhile clear follicular fluid as HFs. When the surfaces appeared gray to white, with fewer capillaries and turbidity in the follicular fluid in conjunction with dark masses inside the follicles or the follicles having severe internal flocculation, these were classified as AFs (34). Then, we detected the hormone content of follicular fluid (estrogen and PROG) by ELISA, the apoptosis of GCs by flow cytometric analysis, and the microstructure of GCs by TEM between HFs and AFs.

### Detection of E_2_ and PROG in Follicular Fluid

The follicular fluid was collected from HFs (*n* = 6) and AFs (*n* = 5), centrifuged for 2,000 ***g***for 10 min, and E_2_ and PROG were measured in the supernatants using ELISA kits (Cusabio, CSB-E08893b, Wuhan, China), and an enzyme-labeled instrument (Tecan Trading AG, Mannedorf, Switzerland) was used for measurements. All assays were validated in our laboratory by showing parallelism between serial sample dilutions and the provided assay standard curves (ranges, 40–1,000 pg/ml for E_2_ and 0.5–30 ng/ml for PROG). Sensitivities of the assays were 40 pg/ml and 0.2 ng/ml, and the intra-assay coefficients of variations were 15% for each.

### Detection of Apoptosis of GCs

The FITC Annexin V Apoptosis Detection Kit I (BD Pharmingen, 556547, Franklin Lakes, NJ, USA) was used to measure apoptosis. GCs were collected and washed twice with cold PBS and then resuspended in 1 × binding buffer at a concentration of 1 × 10^6^ cells/ml. Five microliters of FITC Annexin V and 5 μl PI were added, and the cells were gently vortexed and then incubated for 15 min at room temperature (25°C) in the dark. Four hundred microliters of 1 × binding buffer was then added to each tube per test, and the samples were analyzed by flow cytometry (ACCURI C6, BD, Franklin Lakes, NJ, USA) within 1 h.

### TEM Staining of GCs

GCs were collected and washed twice with cold PBS, and the cell suspension was fixed at 4°C by mixing with an equal volume of fixing solution: 2.5% glutaraldehyde and 0.1 M phosphate buffer as solvent (Servicebio, G1102, Wuhan, China) for 2–4 h. The cells were transferred to centrifuge tubes and spun to get the cell pellets, and then washed three times with 0.1 M PBS. Dehydration was performed by placing the samples in the following solvents for 15 min each: 50% ethanol, 70% ethanol, 80% ethanol, 90% ethanol, 95% ethanol, two changes of 100% ethanol, and two changes of acetone. The samples were then infiltrated with 1:1 acetone: EMbed 812 (SPI, West Chester, PA 90529-77-4, USA) for 2–4 h followed by 2:1 acetone: EMbed 812 overnight in a desiccator without covering and then pure EMbed 812 for 5–8 h in a 37°C oven. Then, the samples were embedded by baking in a 60°C oven for 48 h. Ultrathin sections (60–80 nm) were cut with an ultramicrotome (Leica UC7, Leica, Wetzlar, Germany). Sections were stained with uranyl acetate in pure ethanol for 15 min and then rinsed with distilled water. Then, they were stained with lead citrate for 15 min and again rinsed with distilled water. The sections were air-dried overnight and observed on a TEM (HT7700, Hitachi, Tokyo, Japan).

### RNA Preparation and Sequencing Analysis

Total RNA was extracted from the GCs of HFs and AFs by using the TRIzol™ Reagent (Life Technologies, Mt Waverley, VIC, Australia) according to the manufacturer's protocol. The protocol was as follows: drastic mechanic vibration after add 1 mL of TRIzol® reagent (50–100 mg tissue or 5–10 × 10^6^ cells), add 0.2 ml of chloroform and leave for 2–3 min (room temperature), centrifuge (12,000 × ***g***, 4°C for 15 min), add 0.5 ml of isopropanol and leave for 5–10 min (room temperature), centrifuge (12,000 × ***g***, 4°C for 10 min) for RNA deposition, and then add 1 ml 75% ethanol for RNA washing. RNA quality and integrity were estimated with an Agilent 2100 Bioanalyzer and RNA 6000 Nano Kit Reagents (Agilent Technologies, Santa Clara, CA, USA). Only high-quality RNA samples (concentration ≥ 250 ng/μl, RIN ≥ 7.0, total content ≥ 20 ng) were used to construct the sequencing libraries.

### RNA-Seq

Details of the mRNA-seq, miRNA-seq, and lncRNA-seq methods are described in detail in Supplementary Material.

### CeRNA Regulatory Network

Correlations of expression between mRNAs, miRNAs, and lncRNAs were evaluated using the Spearman rank correlation coefficient (SCC). Pairs with SCC < −0.7 were selected such as those with negatively co-expressed lncRNA–miRNA pairs or mRNA–miRNA pairs, or those where both mRNA and lncRNA were miRNA target genes and also where all the RNAs were differentially expressed. Pairs with Pearson correlation coefficients (PCC) > 0.9 were selected as co-expressed lncRNA–mRNA pairs, where both the mRNAs and lncRNAs in the pair were targeted and co-expressed negatively with a common miRNA. As a result, only the gene pairs with a *p*-value < 0.05 were selected. Pathway significant enrichment analysis used the KEGG pathway as a unit, and the hypergeometric test was used to determine whether the pathway was significantly enriched in differentially expressed transcripts when compared with the background of the whole transcripts.

### Quantitative Real-Time PCR (qRT-PCR) Experimental Validation

qRT-PCR was performed to validate the expression levels of DEmRNAs, DElncRNAs, and DEmiRNAs. Total RNA was extracted by using the TRIzol™ Reagent (Life Technologies, Mt Waverley, VIC, Australia) according to the manufacturer's protocol. First-strand cDNA was synthesized from 1 μg of total RNA with the HiScript® II QRT SuperMix (Vazyme, Cat#R223, Nanjing, China) for qPCR. In addition, 1 μg of small RNA was used for cDNA synthesis using a miRNA 1st-Strand cDNA Synthesis Kit (reaction contains 10 μl of 2 × RT Mix, 2 μl of random hexamers, 2 μl of HiScript II Enzyme Mix, and 1 pg−1 μg total RNA from the samples, added with ddH_2_O to a final volume of 20 μl) (Vazyme, Cat#MR101, China) with the stem-loop primers designed using the stem-loop sequence (GTCGTATCCAGGGTCCGAGGTATTCGCACTGGATACGAC) except for the internal reference, U6. qPCR was performed on the Applied Biosystems™ 7500 Real-Time PCR Systems using 10 μl of ChamQ™ Universal SYBR® qPCR Master Mix (Vazyme, Cat#Q711), lnRcute lncRNA qPCR Detection Kit (Cat#FP402, Tiangen, Beijing, China), and miRNA Universal SYBR® qPCR Master Mix (Vazyme, Cat#MQ101) in each 20-μl reaction volume per well by following the manufacturer's instructions. The 2^−Δ*ΔCT*^ method was used to normalize and determine the RNA level relative to an internal reference gene, beta-actin (Cs1g05000.1) or U6 (35). All primers used are listed in Supplementary Table 3.

### Dual-Luciferase Reporter Assay

PsiCHECK 2.0-*Hif1a* WT and psiCHECK 2.0-*Hif1a* mut 1, 2, and 3 and psiCHECK 2.0-lnc4040 WT and psiCHECK 2.0-lnc4040 mut were constructed and validated by DNA sequencing. 293T cells (5 × 10^4^ per well) were seeded in 24-well plates and grown to a density of 70–80%. Cotransfection of cells was performed using 50 nM miR-709 mimic, NC, and 25 nM miR-709-inhibitor, NC (RiboBio, Guangzhou, China), or a 0.5 μg reporter vector by X-tremeGENE™ HP DNA Transfection Reagent (6366244001, Roche, Basel, Switzerland). Twenty-four hours after transfection, culture medium was removed, cells were gently washed with PBS once, and 100 μl of passive lysis buffer was added and incubated at 37°C for 10–12 min. Cells were lysed by pipetting up and down several times and centrifuged at 5,000 rpm for 4 min to remove debris, and 10–20 μl was used to assay for luciferase activity using dual luciferase reporter assay (GeneCopoeia, LF004, Rockville, MD USA) in a single injector luminometer. The method refers to the previous study (36).

### Vector Construction and Transfection

The lncRNA overexpression vector was constructed using the linear sequence of lnc4040 amplified from GCs by PCR. They were then cloned into the pcDNA3.1-EGFP vector in accordance with the manufacturer's protocol using the NheI and KpnI restriction sites. GCs were collected from follicular fluid and cultured to P2 with 10% FBS (Gibco-BRL, Grand Island, NE, USA) added to normal DMEM (Gibco-BRL, Grand Island, USA). miRNA mimics, mimics NC, and lncRNA overexpression vector were transiently transfected into GCs using Lipofectamine 3000 reagent (Invitrogen, Carlsbad, CA, USA). GCs were collected 48 h after transfection, and proteins were extracted for further experiments.

### Western Blot Analysis

Total proteins from GCs were homogenized using RIPA buffer (Servicebio, G2002, Wuhan, China). Protein concentrations were determined using the BCA Protein Assay Kit (Solarbio, PC0020, Wuhan, China). Proteins were separated by 10% sodium dodecyl sulfate polyacrylamide gel electrophoresis, transferred to a polyvinylidene fluoride membrane (Millipore, IEVH00005, Burlington, MA, USA), and then incubated with antibodies (HIF1A, Novus, NB100, Littleton, CO, USA; GAPDH, Proteintech, 60004-1-Ig, Wuhan, China) overnight at 4°C and then with HRP-conjugated secondary antibody for 1 h at room temperature. Pictures were captured by an imaging system (UVP).

### Statistical Analysis

The quantitative results are presented as means ± SEM based on at least three independent experiments. Significant variance in multiple comparisons was performed using multiple *t*-tests and one-way ANOVA with GraphPad Prism version 8.0 (GraphPad Software, La Jolla, CA, USA). Differences were considered significant when *p*-values ≤ 0.05.

## Discussion

At present, there is no exact method to judge the difference between healthy and atresia-associated buffalo follicles. Previous judgment on the appearance of sheep follicles was reported (37), and this was applied to naked follicles collected and observed in these studies. The hormone levels in follicular fluid were also used as an important basis for judging whether the follicle was in the process of atresia. The ratio of E_2_ to PROG in follicular fluid has been discussed differently in different species. The PROG/E_2_ ratio of ≥10 usually indicated follicle atresia in bovine (38), and the E_2_/PROG ratio of healthy camel follicles was 22.8 while that of AFs was 0.08 (39), and in swine, follicles with a PROG/E_2_ ratio of <5 were always classified as HF (40). Through a comprehensive comparison of the hormone ratios and appearance, it was found that the ratio of E_2_ to PROG in follicular fluid for HFs was 5.77 and that for AFs was 1.43 in our study. Follicular atresia is mainly attributed to GC apoptosis (1, 41–43), and the apoptosis rate of GCs in HFs is basically <10%, while that in AFs can be as high as 30% (44–46). In bovine, the apoptosis rate of follicular GCs with <10% apoptotic cells was designated as HFs (44). In this study, the apoptosis rate of GCs in HFs was 4.93 ± 1.59; that is, the proportion of healthy cells was about 95%, which was consistent with previous studies. The accuracy of sequencing and grouping was guaranteed by the comprehensive determination of three indicators.

In this study, whole-transcriptome RNA sequencing was performed for the first time on buffalo ovarian GCs, and the differences between HFs and AFs at the RNA level were analyzed systematically. Our study found 2,936 DEmRNAs (Supplementary Table 2 for details), among which *Serpine2, Inha, Hif1a, Fst, Jak3, Cyp19a1*, and *Vegfa* were the top ones with the highest expression levels, and these were highly expressed in HF. *Serpine2* was at the highest level in GCs of growing dominant bovine follicles (47). This is in accordance with the report that the expression of *Serpine2* was regulated by follicle-stimulating hormone (FSH) and growth factors in non-luteinizing bovine GCs, and the authors proposed that it could regulate atresia in bovine follicles (48). This is consistent with the high expression of *Serpine2* in AFs, which further indicates that *Serpine2* is needed in GCs of AFs. *In vitro* studies on follicle wall explants confirmed the significant differential expression of *Inha* and *Fst* during follicle development in the laying hen (49). Moreover, several specific mRNAs expressed in GCs during follicular development detected in previous studies were confirmed in this experiment (50–52).

There were also many reports on the regulation of follicular development by miRNA in GCs. A previous study confirmed that miR-21-3p inhibits bovine GC autophagy by targeting VEGFA so as to regulate follicular atresia (53). In addition, miRNA-10001-3p and miRNA-12030-3p were shown to regulate VEGFA and FST, which suggests that both these factors probably act synergistically during buffalo follicular atresia. *Hif1a*, as a regulator of VEGF, has been shown to induce autophagy of follicular GCs *via* FSH (31). In our study, it was found that *Hif1a* expressed highly in HFs, suggesting that it might promote autophagy of GCs in order to maintain the dynamic balance of follicular development and protect the follicles from entering atresia.

One of the uppermost markers of ovarian differentiation, FOXL2, is necessary for the normal development of GCs (30, 54), and its expression was the highest in these cells, followed by stromal cells, and it was not expressed in oocytes (54, 55). It was predicted that miR-212-3p may target FOXL2 in buffalo GCs, and the results of sequencing and quantitative analysis confirmed the high expression of FOXL2 and the low expression of miR-212-3p in HFs. The results seen for SOX9, which has the opposite effect to FOXL2, showed that its expression in AFs was higher than that in HFs and it is a target by miR-424-3p. This finding is important within the context of the regulation of follicular atresia.

By visualizing the network diagram, several pairs of important ceRNA relationships are seen. These include a key miRNA, miR-424-3p, which is predicted to target four DEmRNAs in GCs between HFs and AFs, including FGFR1 (TCONS_00004077), SGK1 (XM_006059282.2), SOX9 (XM_025279387.1), and THBS (XM_025294260.1). In addition, miR-212-3p is predicted to target FOXL2 (XM_025290957.1), JAK3 (TCONS_00098588), and LDHA (XM_006056061.2). Also, miR-709-5p is seen to target *Hif1a* (TCONS_00111150) and INHBB (XM_025277483.1). All these target mRNAs regulate cell proliferation, apoptosis, and response to hormones and play regulatory roles at different stages of follicular development.

The ceRNA hypothesis was first proposed by Pandolf in 2011. He presented a unifying hypothesis regarding how mRNAs, transcribed pseudogenes, and lncRNAs “communicate” to each other using microRNA response elements (MREs) (56). MiRNAs can bind to the target mRNAs, inhibiting their translation or leading to their degradation, thus achieving the function of posttranscriptional regulation of gene expression. In addition to pseudogenes, mRNAs and lncRNAs can function as ceRNAs.

In this study, 49.6% of DEmRNAs were predicted as the targets of 135 DEmiRNAs, which constituted 4,309 DEmiRNA–DEmRNA interactions. Half of the DEmRNAs were not used during construction of the ceRNA network because the SCCs of expression between mRNA and miRNA were < -0.7. Also, the PCC of the targeting relationship with miRNA was >0.9 and the *p*-values were < 0.05. These DEmRNAs competitively bound 134 DElncRNAs as ceRNAs. Because miRNAs play a critical role in the connection and regulation of different ceRNAs, particular attention was paid to the following miRNAs: miR-212-3p, miR-424-3p, and miR-709-5p. MiR-212 had been shown to be highly induced by LH in GCs, thus reducing the expression of CTBP1 protein (57). An increase in follicular levels of miR-212 was also observed following administration of an ovulatory dose of hCG (58). Studies on miR-424 showed that it is associated with therapeutic targets for a variety of diseases, such as muscular dystrophy, liver cancer, HPV, and vascular remodeling as well as regulating the STAT, NOTCH, and HIF1A signaling pathways (59–62).

In our study, XR_003106012.1 and XR_003106717.1 were found to competitively bind to miR-424-3p and TCONS_00104040 was competitively bound to miR-709-5p. It suggests that lncRNA may function as miRNA sponges to participate the regulation of follicular development. However, it should be pointed out that most of DElncRNAs were not included in the ceRNA network. Therefore, it is assumed that these lncRNAs are involved in the regulation of follicular development *via* other mechanisms. These may include a requirement for transcription or splicing of an RNA, due to DNA elements within the lncRNA promoter or gene body that function independently of the transcription, induction of DNA methylation, and modification of chromatin or serve as transcription enhancers (63).

During follicular development, miRNAs act as key regulators to modulate the upregulation or downregulation of important GC processes such as proliferation, apoptosis, differentiation, transcriptional regulation, and modification. As a consequence, through the regulation of GCs, it may be possible to control the development of dominant follicles to mature follicles and ovulate, while the non-dominant or unselected follicles gradually proceed to atresia, so as to regulate the dynamic development of follicles and maintain the balance of ovarian development. In this process, some lncRNAs can act as ceRNAs to competitively bind the MRE of miRNAs, which may indirectly affect the expression of particular mRNAs. Our experimental validation demonstrated that lnc4040 function as miRNA (miR-709) sponges to regulate the abundance of HIF1α. Thus, we proposed that lncRNA can regulate the abundance of key transcription factors in a ceRNA manner, then fine-tune the expression of cell-related genes in GCs during follicle development. However, further work is needed to elucidate their functions in buffalo follicular atresia.

In general, we have carried out an in-depth molecular analysis of GCs in HFs and AFs. Taking the buffalo as an entry point, we can temporarily fill in the gaps in the non-coding RNA data of this species in the field of reproduction. The buffalo could also serve as a model for mono-ovulating mammals, which could be useful for other mono-ovulating livestock. Next studies will investigate the role of non-coding RNAs in regulating the physiological state and function of GCs, thus affecting follicle development and atresia in Chinese buffalo.

## Data Availability Statement

The datasets presented in this study can be found in online repositories. The names of the repository/repositories and accession number(s) can be found in the article/Supplementary Material.

## Ethics Statement

The animal study was reviewed and approved by Animal Care & Welfare Committee of Guangxi University.

## Author Contributions

YP, SY, DS, and YD designed the research. YP, JC, QL, and QX performed the research. YP, RZ, and JL analyzed the data. SY provided the experimental material. YP, DS, and YD wrote the paper. DS and YD provided the supervision and funding. All authors contributed to the article and approved the submitted version.

## Conflict of Interest

The authors declare that the research was conducted in the absence of any commercial or financial relationships that could be construed as a potential conflict of interest.
