# Supplementary material for: Whole-Transcriptome Analysis of LncRNAs Mediated ceRNA Regulation in Granulosa Cells Isolated From Healthy and Atresia Follicles of Chinese Buffalo
Source: Front Vet Sci. 2021 Jul 14;8:680182. doi: 10.3389/fvets.2021.680182 (PMC8316591; doi:10.3389/fvets.2021.680182)
Supplement: Supplementary file 3 [file Table_3.DOCX]

Table S3 primers for QRT-PCR

| GENE ID | primers |
| --- | --- |
| TCONS_00111150-F | ACTTTTGGGCCGCTCAATTT |
| TCONS_00111150-R | CCACCTCTTTTGGCAAGCAT |
| TCONS_00104040-F | TGTAAGATGGGACTCAGAGA |
| TCONS_00104040-R | ACTTCTCTAAAATCACCCCC |
| miR-709-F | AGGCTGAGGCTGG |
| miR-709-RT | GTCGTATCCAGTGCGTGTCGTGGAGTCGGCAATTGCACTGGATACGACATCCTCC |
| miR-X-R | GTGCAGGGTCCGAGGT |
| U6-F | CTCGCTTCGGCAGCACA |
| U6-R | AACGCTTCACGAATTTGCGT |
| U6-RT | CGCTTCACGAATTTGCGTGTCAT |
|  |  |
