# Supplementary material for: Whole-Transcriptome Analysis of LncRNAs Mediated ceRNA Regulation in Granulosa Cells Isolated From Healthy and Atresia Follicles of Chinese Buffalo
Source: Front Vet Sci. 2021 Jul 14;8:680182. doi: 10.3389/fvets.2021.680182 (PMC8316591; doi:10.3389/fvets.2021.680182)
Supplement: Supplementary file 5 [file Data_Sheet_1.docx]

mRNA&lncRNA-seq

# Flow chart of Experiments

Total RNA

Remove rRNA

RNA fragmentation (200-500nt)

First strand cDNA synthesis

Second strand cDNA

synthesis

Ligate adapter

UNG treatment

PCR amplification

Sequencing

Fig 1.1 Flow chart of long non-coding RNA experiments


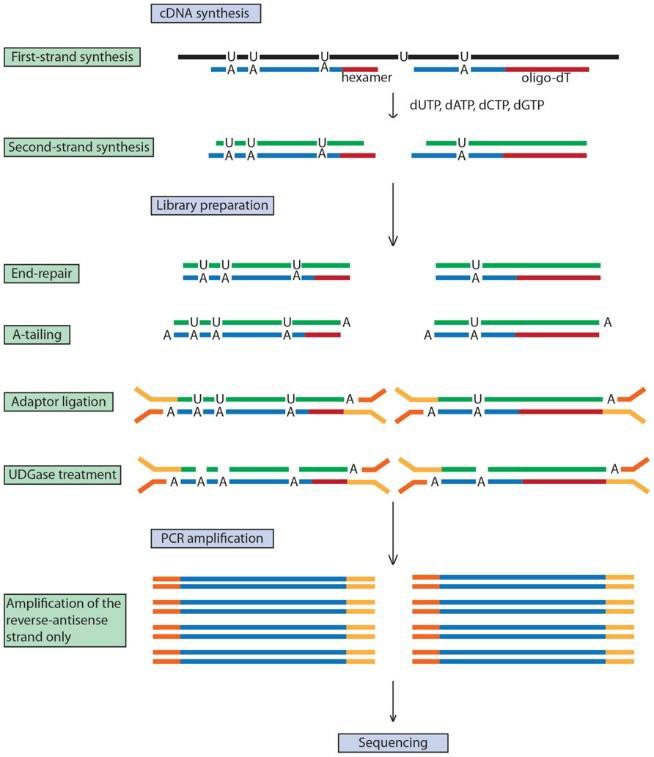


Fig 1.2 Flow chart of strand-specific library construction experiments

Strand-specific library construction and sequencing

After total RNA was extracted, rRNAs were removed to retain mRNAs and ncRNAs. The enriched mRNAs and ncRNAs were fragmented into short fragments by using fragmentation buffer and reverse transcripted into cDNA with random primers. Second- strand cDNA were synthesized by DNA polymerase I, RNase H, dNTP (dUTP instead of dTTP) and buffer. Next, the cDNA fragments were purified with QiaQuick PCR extraction kit, end repaired, poly(A) added, and ligated to Illumina sequencing adapters. Then UNG (Uracil-N-Glycosylase) was used to digest the second-strand cDNA. The digested products were size selected by agarose gel electrophoresis, PCR amplified, and sequenced using Illumina HiSeqTM 4000 (or other platforms) by Gene Denovo Biotechnology Co. (Guangzhou, China).

#
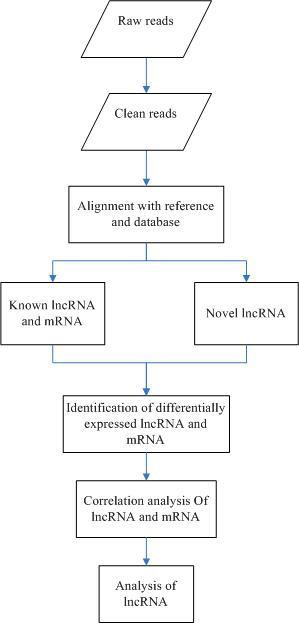
Flow chart of bioinformatics analysi

Fig 2.Flow chart of bioinformatics analysis

# Filtering of Clean Reads

Reads obtained from the sequencing machines included raw reads containing adapters or low quality bases which would affect the following assembly and analysis. Thus, to get high quality clean reads, reads were further filtered according to the following rules:

1. Removing reads containing adapters;
2. Removing reads consisting of all A bases;
3. Removing reads containing more than 10% of unknown nucleotides (N);
4. Removing low quality reads containing more than 50% of low quality (Q-value≤20) bases.

# Alignment with Ribosome RNA (rRNA)

Short reads alignment tool Bowtie2 (2.2.8)[1] was used for mapping reads to ribosome RNA

(rRNA) database. The rRNA mapped reads were then removed. The remaining reads were further used in assembly and analysis of transcriptome.

# Alignment with reference genome

The rRNA removed reads of each sample were then mapped to reference genome by TopHat2

[2] (version 2.1.1), respectively. The alignment parameters were as follows:

1. Maximum read mismatch is 2
2. Disables the coverage based search for junctions
3. The distance between mate-pair reads is 50bp
4. The standard deviation for the distribution on inner distance between mate-pair reads is 80bp

After aligned with reference genome, unmapped reads (or mapped very poorly) were then re-aligned with Bowtie2, the enriched unmapped reads were split into smaller segments which were then used to find potential splice sites. The section and the section position of these short segments were predicted as well. A set of splice sites was built with initial unmapped reads by TopHat2 without relying on the known genes annotation[3]. Not only be used for identifying expressed genes and their quantitative expression, the sequence alignment will also be helpful to find alternative splicing and new transcripts.


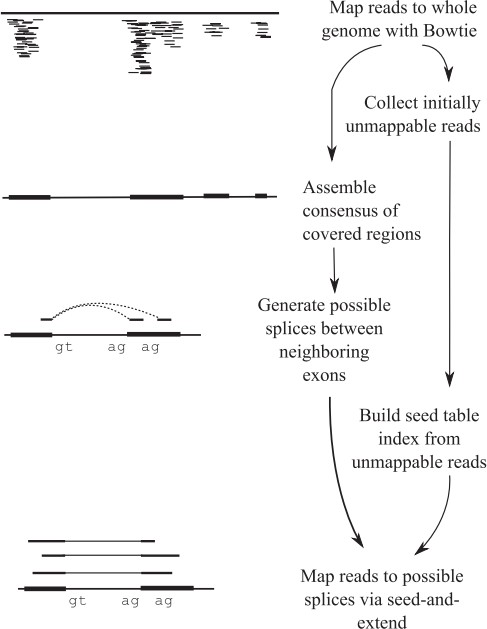


Fig 3. Flow chart of Tophat2

# Transcripts Reconstruction

The reconstruction of transcripts was carried out with software Cufflinks [4], which together with TopHat2, allow biologists to identify new genes and new splice variants of known ones. The program reference annotation based transcripts (RABT) was preferred. Cufflinks constructed faux reads according to reference to make up for the influence of low coverage sequencing. During the last step of assembly, all of the reassembles fragments were aligned with reference genes and then similar fragments were removed.


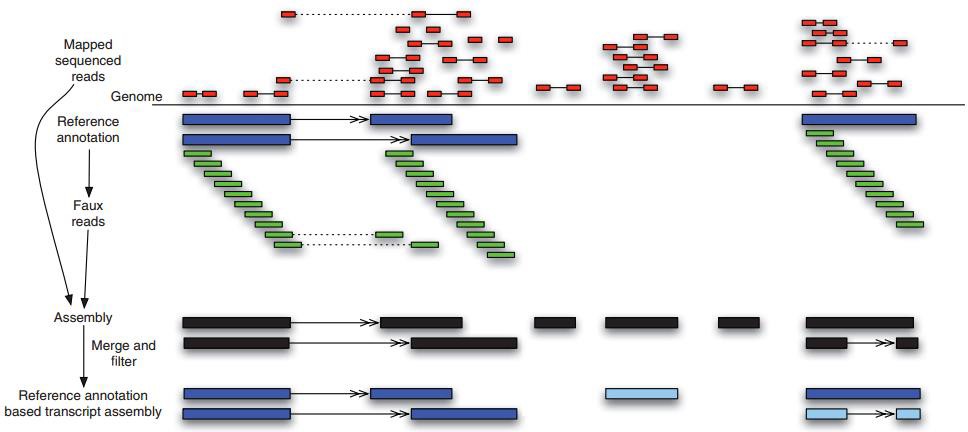


Fig 4. Flow chart of RABT Assembly


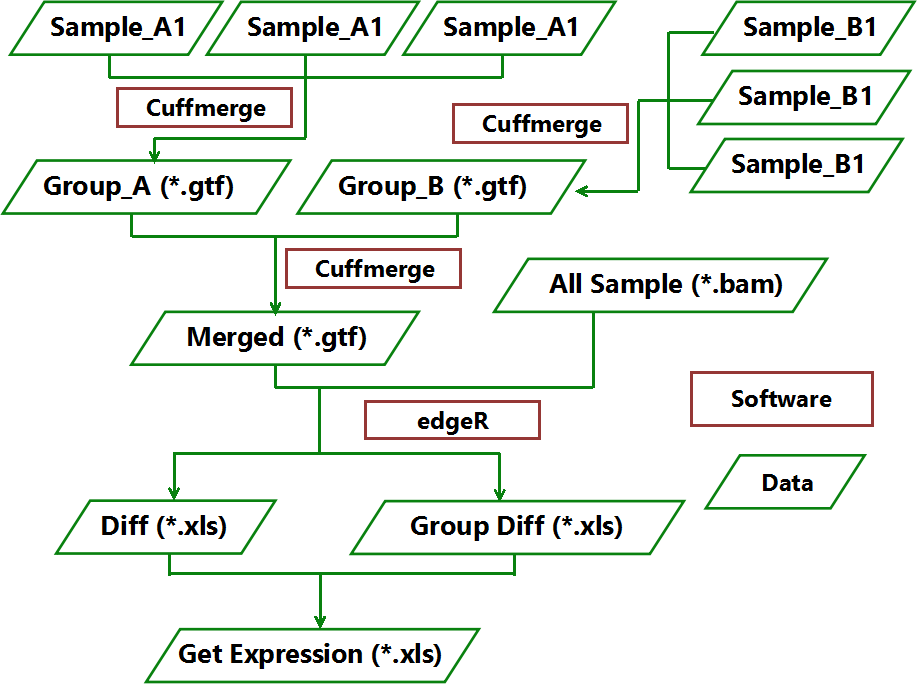
Then we used Cuffmerge to merge transcripts from different replicas of a group into a comprehensive set of transcripts, and then merge the transcripts from multiple groups into a finally comprehensive set of transcripts for further downstream differential expression analysis.

Fig 5. Multiple samples differential expression

# Novel Transcripts Identification and Annotation

To identify the new transcripts, all of the reconstructed transcripts were aligned to reference genome and were divided into twelve categories by using Cuffcompare. Transcripts with one of the classcodes “u,i,j,x,c,e,o” were defined as novel transcripts. We used the following parameters to identify reliable novel genes:

the length of transcript was longer than 200bp and the exon number was more than 2 (plants more than 1).

Novel transcripts were then aligned to the Nr, KEGG, and GO database to obtain protein functional annotation.

# lncRNA Prediction

Two softwares CNCI (version 2)[5] and CPC[6]( <http://cpc.cbi.pku.edu.cn/)>were used to assess

the protein-coding potential of novel transcripts by default parameters. In the mean time, novel transcripts were mapped to SwissProt database to assess protein annotation. The intersection of both non protein-coding potential results and non protein annotation results were chosen as long non-coding RNAs.

# lncRNA type classification

lncRNAs were classified into five classes according to their location relative to protein-coding genes: intergenic lncRNAs, bidirectional lncRNAs, intronic lncRNAs, antisense lncRNAs, and sense overlapping lncRNAs. Different types of lncRNAs may have different biological functions.

# Quantification of Transcripts Abundance

Transcripts abundances were quantified by software RSEM[7]. There were two steps for RSEM to quantify transcripts abundances. Firstly, a set of reference transcript sequences were generated and preprocessed according to known transcripts and new transcripts (in FASTA format) and gene annotation files (in GTF format). Secondly, reads were realigned to the reference transcripts by Bowtie alignment program and the resulting alignments were used to estimate transcript abundances.

The transcript expression level was normalized by using FPKM (Fragments Per Kilobase of transcript per Million mapped reads) method, and the formula is shown as follows:


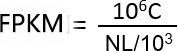


Given FPKM(A) to be the expression of transcripts A, C to be number of fragments mapped to transcripts A, N to be total number of fragments that mapped to reference genes, and L to be number of bases on transcripts A. The FPKM method is able to eliminate the influence of different transcripts lengths and sequencing data amount on the calculation of transcripts expression. Therefore, the calculated transcripts expression can be directly used for comparing the difference of transcripts expression among samples.

# Relationship analysis of samples

- - 1. Correlation Analysis of Replicas

Correlation analysis of two parallel experiments provides the evaluation of the reliability of experimental results as well as operational stability. The correlation coefficient between two replicas was calculated to evaluate repeatability between samples. The closer the correlation coefficient gets to 1, the better the repeatability between two parallel experiments.

- - 1. Principal Component Analysis

Principal component analysis (PCA) was performed with R package gmodels ([http://www.r-](http://www.r-project.org/) [project.org/](http://www.r-project.org/)) in this experience. PCA is a statistical procedure that converts hundreds of thousands of correlated variables (transcripts expression) into a set of values of linearly uncorrelated variables called principal components. PCA is largely used to reveal the structure/relationship of the samples/datas.

# Differentially expressed transcripts (DEGs) Analysis

The differentially expressed transcripts of coding RNAs and lncRNAs were analyzed respectively. To identify differentially expressed transcripts across samples or groups, the edgeR package (<http://www.r-project.org/>) was used. We identified transcripts with a fold change ≥2 and a false discovery rate (FDR) <0.05 in a comparison as significant DEGs. Differentially expressed coding RNAs were then subjected to enrichment analysis of GO functions and KEGG pathways.

- - 1. GO Enrichment Analysis

Gene Ontology (GO) is an international standardized gene functional classification system which offers a dynamic-updated controlled vocabulary and a strictly defined concept to comprehensively describe properties of genes and their products in any organism. GO has three ontologies: molecular function, cellular component and biological process. The basic unit of GO is GO-term. Each GO-term belongs to a type of ontology.

GO enrichment analysis provides all GO terms that significantly enriched in DEGs comparing to the genome background, and filter the DEGs that correspond to biological functions. Firstly all DEGs were mapped to GO terms in the Gene Ontology database [(h](http://www.geneontology.org/))t[tp://www.geneontology.org/),](http://www.geneontology.org/)) gene numbers were calculated for every term, significantly enriched GO terms in DEGs comparing to the genome background were defined by hypergeometric test. The calculating formula of P-value is:


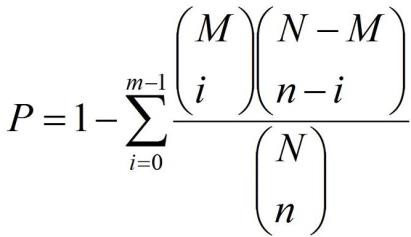


Here N is the number of all genes with GO annotation; n is the number of DEGs in N; M is the number of all genes that are annotated to the certain GO terms; m is the number of DEGs in

M. The calculated p-value were gone through FDR Correction, taking FDR ≤ 0.05 as a threshold. GO terms meeting this condition were defined as significantly enriched GO terms in DEGs. This analysis was able to recognize the main biological functions that DEGs exercise.

- - 1. Pathway Enrichment Analysis

Genes usually interact with each other to play roles in certain biological functions. Pathway- based analysis helps to further understand genes biological functions. KEGG is the major public pathway-related database[8]. Pathway enrichment analysis identified significantly enriched metabolic pathways or signal transduction pathways in DEGs comparing with the whole genome background. The calculating formula is the same as that in GO analysis.


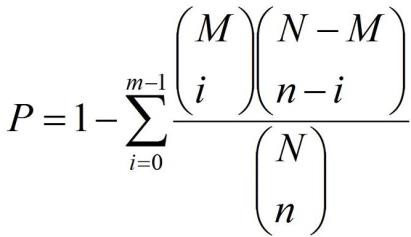


Here N is the number of all transcripts that with KEGG annotation, n is the number of DEGs in N, M is the number of all transcripts annotated to specific pathways, and m is number of DEGs in M. The calculated p-value was gone through FDR Correction, taking FDR ≤ 0.05 as a threshold. Pathways meeting this condition were defined as significantly enriched pathways in DEGs.

# lncRNA Family Analysis

Rfam is an annotated open access database containing information about non-coding RNA (ncRNA) families, consensus RNA secondary structures and other structured RNA elements. Rfam divides ncRNAs into families based on evolution from a common ancestor. Producing multiple sequence alignments of these families can provide insight into their structure and function, similar to the case of protein families. To better annotate lncRNA in evolution level, the software Infernal[9]（<http://eddylab.org/infernal/>） was used in sequence alignment. lncRNAs were classified by secondary structures and sequence conservation.

# miRNA precursor Prediction

lncRNAs can be spliced into multiple small RNAs which function as post-transcriptional regulators. To find potential miRNA precursors, lncRNAs were aligned to miRBase（version 21）. Those with identity more than 90% were selected. In addition, the software miRPara[10], which is based on SVM method was also used to predict miRNA precursors.

# Gene Structure Optimization

The gene structure and annotation for model organisms such as people, mice and Arabidopsis thaliana are almost complete, but for other species, the reads can be used to optimize their gene structure and annotation. After mapping reads to reference genome, the Cufflink was used in reconstruction of transcripts which may extend the 5' untranslated region (5’UTR) or 3’UTR of gene as optimizing the gene structure.

# Single-nucleotide Polymorphism (SNP) Analysis

The GATK [11] was used for calling variants of transcripts, and ANNOVAR[12] was used for SNP/InDel annotation. The function, genome site and type of variation of SNPs were also analyzed.

RNA editing:

RNA editing refers to variants arising from mRNA transcription.

We used the following criteria to screen reliable editing sites from SNP sites [13] [14]:

1. Removing the low quality SNPs while calling SNP by GATK.
2. Correcting the SNPs around InDel region.
3. Choosing non-overlapping SNPs in UTR and EXON region.
4. Choosing SNPs with reference reads>=2 and variant reads>=3.
5. Choosing SNPs with the variation frequency between 0.1 and 0.9.

# Alternative Splicing Analysis

The results of Tophat included all alternative splicing information and the junction structure is shown as follows:


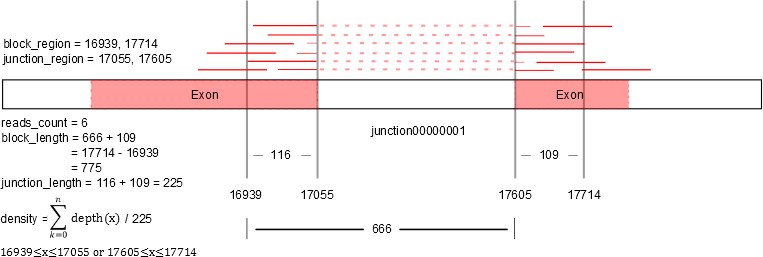


Fig 6. Structure of Junction

rMATS (<http://rnaseq-mats.sourceforge.net/index.html>) was used to identify alternative splicing events and analyze differential alternative splicing events between samples. We identified AS events with a false discovery rate (FDR) <0.05 in a comparison as significant AS events.

The classification of alternative splicing is as follows:

- SE: skipped exon
- MXE: mutually exclusive exon
- A5SS: alternative 5’ splice site
- A3SS: alternative 3’ splice site
- RI: retained intron

# lncRNA-mRNA association analysis

- - 1. Antisense lncRNA Analysis

LncRNAs are involved in many post-transcriptional regulation processes, same as some other small RNAs like miRNA and snoRNA, those regulations are always related with complementary base pairing. Some antisense lncRNAs may regulate gene silencing 、transcription and mRNA stability. In order to reveal the interaction between antisense lncRNA and mRNA, the software RNAplex[15] [(h](http://www.tbi.univie.ac.at/RNA/RNAplex.1.html)t[tp://www.tbi.univie.ac.at/RNA/RNAplex.1.html](http://www.tbi.univie.ac.at/RNA/RNAplex.1.html) ) was used to predict the complementary correlation of antisense lncRNA and mRNA. The program contains ViennaRNA package, and the prediction of best base pairing was based on the calculation of minimum free energy through thermodynamics structure.

- - 1. lncRNA cis-regulation Analysis

One of the functions of lncRNAs is cis-regulation of their neighboring genes on the same allele. The up-stream lncRNAs which have intersection of promoter or other cis-elements may regulate gene expression in transcriptional or post-transcriptional level. The down- stream or 3’UTR region lncRNAs may have other regulatory functions. Thus lncRNAs which had been previously annotated as “unknown region” were annotated again. lncRNAs in less than 100kb up/down stream of a gene were likely to be cis-regulators. The cis target genes were then subjected to enrichment analysis of GO functions and KEGG pathways.


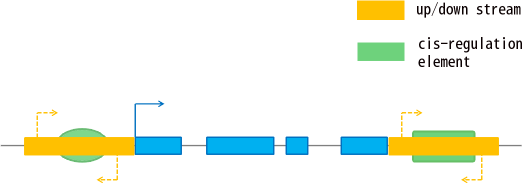


Fig 7. Cis-regulation Analysis

- - 1. lncRNA trans-regulation Analysis

Another function of lncRNAs is trans-regulation of co-expressed genes not adjacent to lncRNAs. We analysed the correlation of expression between lncRNAs and protein-coding genes to identify target genes of lncRNAs. Pearson correlation coefficient was used for samples> = 6, and protein-coding genes with absolute correlation more than 0.9 were then subjected to enrichment analysis of GO functions and KEGG pathways. For samples >=24, WGCNA was used to cluster genes with similar expression pattern and construct gene co- expression network. Then enrichment analysis of GO functions and KEGG pathways were conducted in protein-coding genes in the network. Trans-regulation analysis would not be recommended if samples < 6.

4. Reference

1. Langmead B, Salzberg S L. Fast gapped-read alignment with Bowtie 2[J]. Nature methods, 2012, 9(4): 357-359.
2. Kim D, Pertea G, Trapnell C, et al. TopHat2: accurate alignment of transcriptomes in the presence of insertions, deletions and gene fusions[J]. Genome biology, 2013, 14(4): R36.
3. Trapnell C, Williams B A, Pertea G, et al. Transcript assembly and quantification by RNA- Seq reveals unannotated transcripts and isoform switching during cell differentiation[J]. Nature biotechnology, 2010, 28(5): 511-515.
4. Trapnell C, Roberts A, Goff L, et al. Differential gene and transcript expression analysis of RNA-seq experiments with TopHat and Cufflinks[J]. Nature protocols, 2012, 7(3): 562-578.
5. Liang Sun, Haitao Luo, Dechao Bu, et al. Utilizing sequence intrinsic composition to classify protein-coding and long non-coding transcripts. Nucleic Acids Research (2013), doi: 10.1093/nar/gkt646.
6. Kong L, Zhang Y, Ye Z Q, et al. CPC: assess the protein-coding potential of transcripts using sequence features and support vector machine[J]. Nucleic acids research, 2007, 35(suppl 2): W345-W349.
7. Li B, Dewey C N. RSEM: accurate transcript quantification from RNA-Seq data with or without a reference genome[J]. BMC bioinformatics, 2011, 12(1): 1.
8. Kanehisa, M., M. Araki, et al. KEGG for linking genomes to life and the environment. Nucleic Acids Res. 2008.36 (Database issue): D480-4.
9. E. P. Nawrocki and S. R. Eddy, Infernal 1.1: 100-fold faster RNA homology searches, Bioinformatics 29:2933-2935 (2013).
10. Wu Y, Wei B, Liu H, et al. MiRPara: a SVM-based software tool for prediction of most probable microRNA coding regions in genome scale sequences[J]. BMC bioinformatics, 2011,

12(1): 1.

1. Van der Auwera GA, et al. From FastQ Data to High-Confidence Variant Calls: The Genome Analysis Toolkit Best Practices Pipeline. CURRENT PROTOCOLS IN BIOINFORMATICS 2013 43:11.10.1-11.10.33
2. Wang K, Li M, Hakonarson H. ANNOVAR: Functional annotation of genetic variants from next-generation sequencing data. Nucleic Acids Research, 38:e164, 2010
3. Gokul R., Rui Z., Robert P. Identifying RNA editing sites using RNA sequencing data alone. Nat Methods. 2013 February; 10(2): 128–132.
4. Jae Hoon B., Jae-Hyung L., Gang Li. Accurate identification of A-to-I RNA editing in human by transcriptome sequencing. Genome Res. 2012 22: 142-150
5. Tafer H, Hofacker I L. RNAplex: a fast tool for RNA–RNA interaction search[J]. Bioinformatics, 2008, 24(22): 2657-2663.

smallRNA-seq

#
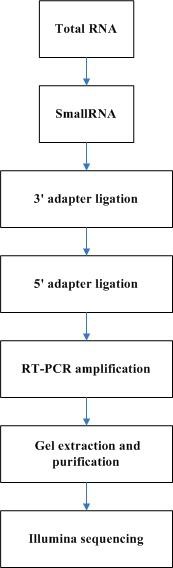
Flow chart of Experiments

Fig 1. Flow chart of smallRNA experiments

Library construction and sequencing

After total RNA was extracted by TRIzol, the RNA molecules in a size range of 18–30nt were enriched by polyacrylamide gel electrophoresis(PAGE). Then the 3’ adapters were added and the 36-44nt RNAs were enriched. The 5’ adapters were then ligated to the RNAs as well. The ligation products were reverse transcripted by PCR amplification and the 140-160bp size PCR products were enriched to generate a cDNA library and sequenced using Illumina HiSeqTM 2500 by Gene Denovo Biotechnology Co. (Guangzhou, China).

#
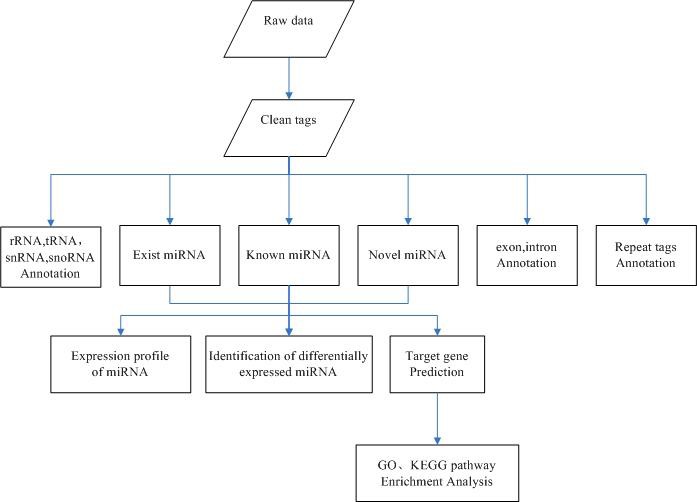
Flow chart of bioinformatics analysis

Fig 2. Flow chart of bioinformatics analysis

# Filtering of Clean Tags

Reads obtained from the sequencing machines included dirty reads containing adapters or low quality bases which would affect the following assembly and analysis. Thus, to get clean tags, raw reads were further filtered according to the following rules:

1. Removing low quality reads containing more than one low quality (Q-value≤20) base or containing unknown nucleotides(N);
2. Removing reads without 3’adapters;
3. Removing reads containing 5’adapters;
4. Removing reads containing 3’ and 5’ adapters but no small RNA fragment between them;
5. Removing reads containing ployA in small RNA fragment;
6. Removing reads shorter than 18nt (not include adapters).

# Alignment and Identification of small RNA

- 1. Alignment with small RNA in GeneBank

All of the clean tags were aligned with small RNAs in GeneBank database(Release 209.0) to identify and remove rRNA,scRNA,snoRNA,snRNA and tRNA.

# Alignment with small RNA in Rfam

Meanwhile all of the clean tags were aligned with small RNAs in Rfam database(11.0) to identify and remove rRNA,scRNA,sonRNA,snRNA and tRNA.

# Alignment with Genome (exon, intron, repeat sequences)

All of the clean tags were also aligned with reference genome. Those mapped to exons or introns might be fragments from mRNA degradation, so these tags were removed. The tags mapped to repeat sequences were also removed.

# Identification of microRNA (miRNA)

- - 1. Identification of exist miRNA

All of the clean tags were then searched against miRBase database(Release 21) to identify known (所研究物种) miRNAs (exist miRNAs).

# Identification of known miRNA

So far the miRNA sequences of some species were still not included in miRBase database. For those species the miRNAs alignment with other species was a dependable way to identify the known miRNAs.

# Identification of novel miRNA

All of the unannotated tags were aligned with reference genome. According to their genome positions and hairpin structures predicted by software Mireap_v0.2, the novel miRNA candidates were identified.

The default parameters of software Mireap_v0.2 were as follows:

1. For animals:
2. Minimal miRNA sequence length is 18nt
3. Maximal miRNA sequence length is 26nt
4. Minimal miRNA reference sequence length is 20nt
5. Maximal miRNA reference sequence length is 24nt
6. Minimal depth of Drosha/Dicer cutting site is 3
7. Maximal copy number of miRNAs on reference is 20
8. Maximal free energy allowed for a miRNA precursor is 18 kcal/mol
9. Maximal space between miRNA and miRNA* is 35nt
10. Minimal space between miRNA and miRNA* is 14nt
11. Maximal bulge between miRNA and miRNA* is 4nt
12. Maximal asymmetry of miRNA/miRNA* duplex is 5nt
13. Flank sequence length of miRNA precursor is 10nt
14. For plans:
15. Minimal miRNA sequence length is 18nt
16. Maximal miRNA sequence length is 25nt
17. Minimal miRNA reference sequence length is 20nt
18. Maximal miRNA reference sequence length is 23nt
19. Maximal copy number of miRNAs on reference is 20
20. Maximal free energy allowed for a miRNA precursor is 18 kcal/mol
21. Maximal space between miRNA and miRNA* is 300nt
22. Minimal space between miRNA and miRNA* is 16nt
23. Maximal bulge between miRNA and miRNA* is 4nt
24. Maximal asymmetry of miRNA/miRNA* duplex is 4nt
25. Flank sequence length of miRNA precursor is 20nt

# Small RNA annotation summary

After tags were annotated as mentioned previously, the annotation results were determined in this priority order: rRNA etc > exist miRNA > exist miRNA edit > known miRNA > repeat > exon > novel miRNA > intron .The tags that cannot be annotated as any of the above molecules were recorded as unann.

# miRNA expression profiles

Total miRNA consists of exist miRNA, known miRNA and novel miRNA, based on their expression in each sample, the miRNA expression level was calculated and normalized to transcripts per million (TPM).

The formula is as follows:

TPM=Actual miRNA counts/Total counts of clean tags*106

In addition, the expression of exist miRNA, known miRNA and novel miRNA was also analysed individually.

Meantime, the analysis of miRNA families was used to identify if the miRNAs were exist in other species. The analysis result was marked as “+” or “-” which refered to exist or non-exist, respectively.

# miRNA Principal Component Analysis

Correlation analysis of two parallel experiments provides the evaluation of the reliability of experimental results as well as operational stability. The correlation coefficient between two replicas was calculated to evaluate repeatability between samples. The closer the correlation coefficient gets to 1, the better the repeatability between two parallel experiments.

# miRNA Expression Pattern Clustering Analysis

The heatmaps of exist miRNA, known miRNA and novel miRNA were drew to display miRNA expression levels in different samples and to cluster miRNAs with similar expression pattern.

# Differentially expressed miRNA (DE miRNA) Analysis


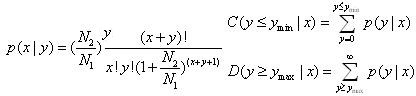
To identify differentially expressed miRNAs across samples or groups the formula was shown as follows:

We identified miRNAs with a fold change ≥2 and P value <0.05 in a comparison as significant DE miRNAs.

# Target gene Prediction

Based on the sequences of the exist miRNAs, known miRNAs and novel miRNAs, the candidate target genes were predicted as follows:

1. For animal samples, three softwares RNAhybrid(v2.1.2)+svm_light(v6.01), Miranda(v3.3a) and TargetScan(Version:7.0) were used to predict targets. The intersection of the results were more credible to be chosen as predicted miRNA target genes.

The default parameters of software RNAhybrid were as follows:

1. Forces structures to have a helix from position 2 to 8 with respect to the query
2. The number of hits per target is 1
3. Maximal bulge loop size is 3nt
4. Maximal internal loop size (per side) is 3nt
5. The cut-off P-value is 0.05
6. The cut-off energy is -10 kcal/mol
7. Maximal query length is 24nt

The default parameters of software Miranda were as follows:

1. The set score threshold is 140
2. The set energy threshold is -10 kcal/mol
3. Demand strict 5’ seed pairing
4. The gap-open penalty is -4.0
5. The gap-extend penalty is -9.0

The default parameters of software TargetScan were as follows:

The 2-8nt sequences which start from 5’ small RNA were choose as seed sequences to predict with 3’-UTR of transcripts.

1. For plant samples, the software patmatch(v1.2) was used to predict target genes. The default parameters were as follows:
2. No more than four mismatches between sRNA \& target (G-U bases count as 0\.5 mismatches)
3. No more than two adjacent mismatches in the miRNA/target duplex
4. No adjacent mismatches in in positions 2\-12 of the miRNA\/target duplex (5\' of miRNA)
5. No mismatches in positions 10-11 of miRNA/target duplex
6. No more than 2\.5 mismatches in positions 1\-12 of the of the miRNA/target duplex (5\' of miRNA)
7. Minimum free energy (MFE) of the miRNA\/target duplex should be \>\= 74% of the MFE of the miRNA bound to it\'s perfect complement

# Target gene functional enrichment Analysis

- 1. GO Enrichment Analysis

Gene Ontology (GO) is an international standardized gene functional classification system which offers a dynamic-updated controlled vocabulary and a strictly defined concept to comprehensively describe properties of genes and their products in any organism. GO has three ontologies: molecular function, cellular component and biological process. The basic unit of GO is GO-term. Each GO-term belongs to a type of ontology.


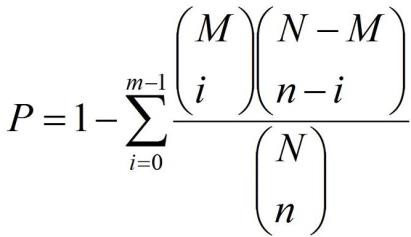
GO enrichment analysis provides all GO terms that significantly enriched in DEGs comparing to the genome background, and filter the DEGs that correspond to biological functions. Firstly all DEGs were mapped to GO terms in the Gene Ontology database [(http://ww](http://www.geneontology.org/))w[.geneontology.org/),](http://www.geneontology.org/)) gene numbers were calculated for every term, significantly enriched GO terms in DEGs comparing to the genome background were defined by hypergeometric test. The calculating formula of P-value is:

Here N is the number of all genes with GO annotation; n is the number of DEGs in N; M is the number of all genes that are annotated to the certain GO terms; m is the number of DEGs in M. The calculated p-value were gone through FDR Correction, taking FDR ≤ 0.05 as a threshold. GO terms meeting this condition were defined as significantly enriched GO terms in DEGs. This analysis was able to recognize the main biological functions that DEGs exercise.

# Pathway Enrichment Analysis


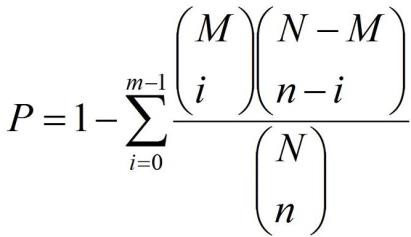
Genes usually interact with each other to play roles in certain biological functions. Pathway-based analysis helps to further understand genes biological functions. KEGG is the major public pathway-related database [9]. Pathway enrichment analysis identified significantly enriched metabolic pathways or signal transduction pathways in DEGs comparing with the whole genome background. The calculating formula is the same as that in GO analysis.

Here N is the number of all genes that with KEGG annotation, n is the number of DEGs in N, M is the number of all genes annotated to specific pathways, and m is number of DEGs in M. The calculated p-value was gone through FDR Correction, taking FDR ≤ 0.05 as a threshold. Pathways meeting this condition were defined as significantly enriched pathways in DEGs. The functional enrichment of both target genes of miRNAs in single samples and DE miRNAs in a compare group were carried out in our analysis.

# 9. Reference

1. Cock P., et al. The Sanger FASTQ file format for sequences with quality scores, and the Solexa/Illumina FASTQ variants. Nucleic Acids Research, 38(6):1767-1771
2. Langmead B, Trapnell C, Pop M, et al. Ultrafast and memory-efficient alignment of short DNA sequences to the human genome[J]. Genome Biol, 2009, 10(3): R25.
3. Hafner, M., P. Landgraf, et al. (2008). "Identification of microRNAs and other small regulatory RNAs using cDNA library sequencing." Methods 44(1): 3-12.
4. Ruby, J. G., C. Jan, et al. (2006). "Large-scale sequencing reveals 21U-RNAs and additional microRNAs and endogenous siRNAs in C. elegans." Cell 127(6): 1193-207.
5. Calabrese, J. M., A. C. Seila, et al. (2007). "RNA sequence analysis defines Dicer's role in mouse embryonic stem cells". Proc Natl Acad Sci USA 104(46): 18097-102.
6. Zhang, Y., X. Zhou, et al. (2009). "Insect-Specific microRNA Involved in the Development of the Silkworm Bombyx mori." PLoS One 4(3): e4677.
7. Allen, E., Z. Xie, et al. (2005). "microRNA-directed phasing during trans-acting siRNA biogenesis inplants." Cell 121(2): 207-21.
8. Schwab, R., J. F. Palatnik, et al. (2005). "Specific effects of microRNAs on the plant transcriptome." Dev Cell 8(4): 517-27.
9. Kanehisa, M., M. Araki, et al. (2008). "KEGG for linking genomes to life and the environment." Nucleic Acids Res. 36 (Database issue): D480-4.
